# Response to Letter to the Editor from Chee et al: “Prevention of Adrenal Crisis: Cortisol Response to Major Stress Compared to Stress Dose Hydrocortisone Delivery”

**DOI:** 10.1210/clinem/dgaa719

**Published:** 2020-10-07

**Authors:** Alessandro Prete, Angela E Taylor, Irina Bancos, David J Smith, Mark A Foster, Sibylle Kohler, Violet Fazal-Sanderson, John Komninos, Donna M O’Neil, Dimitra A Vassiliadi, Christopher J Mowatt, Radu Mihai, Joanne L Fallowfield, Djillali Annane, Janet M Lord, Brian G Keevil, John A H Wass, Niki Karavitaki, Wiebke Arlt

**Affiliations:** 1 Institute of Metabolism and Systems Research, University of Birmingham, Birmingham, UK; 2 Centre for Endocrinology, Diabetes and Metabolism, Birmingham Health Partners, Birmingham, UK; 3 Division of Endocrinology, Metabolism and Nutrition, Department of Internal Medicine, Mayo Clinic, Rochester, MN, USA; 4 School of Mathematics, University of Birmingham, Birmingham, UK; 5 Institute of Inflammation and Ageing, University of Birmingham, Birmingham, UK; 6 NIHR Surgical Reconstruction and Microbiology Research Centre, Queen Elizabeth Hospital, Birmingham, UK; 7 Royal Centre for Defence Medicine, Queen Elizabeth Hospital, Birmingham, UK; 8 Oxford Centre for Diabetes, Endocrinology and Metabolism, Churchill Hospital, Oxford, UK; 9 Department of Endocrinology, Diabetes and Metabolism, Evangelismos Hospital, Athens, Greece; 10 Department of Anaesthesiology, Royal Shrewsbury Hospital, The Shrewsbury and Telford Hospital NHS Trust, Shrewsbury, UK; 11 Department of Endocrine Surgery, Churchill Hospital, Oxford, UK; 12 Institute of Naval Medicine, Alverstoke, UK; 13 Critical Care Department, Hôpital Raymond-Poincaré, Laboratory of Infection & Inflammation U1173 INSERM/University Paris Saclay-UVSQ, Garches, France; 14 NIHR Birmingham Biomedical Research Centre, University of Birmingham and University Hospitals Birmingham NHS Foundation Trust, Birmingham, UK; 15 Department of Clinical Biochemistry, University Hospital of South Manchester, Manchester Academic Health Science Centre, The University of Manchester, Manchester, UK

Chee et al (1) enquired about whether we had gathered clinical data on blood pressure or intraoperative hemodynamic instability in the patients undergoing elective surgery. However, our study (2) looked at cortisol responses to major stress in patients with otherwise normal adrenal function, including healthy patients undergoing elective surgery as well as unstressed controls, soldiers exposed to deployment stress, and patients with severe sepsis. Those were compared to serum cortisol concentrations observed after 4 different modes of hydrocortisone administration in patients with primary adrenal insufficiency.

A clinical study in patients with primary adrenal insufficiency aiming to compare the effects of continuous vs intermittent hydrocortisone delivery on hemodynamic parameters during elective surgery would be very challenging to execute and, in our opinion, only of theoretical benefit. There is no robust clinical evidence that short-term administration of stress dose hydrocortisone for prevention of adrenal crisis has significant adverse effects, while the potentially fatal consequences of glucocorticoid underreplacement in a stressed patient with adrenal insufficiency, in particular when paired with inflammation, are obvious. We think safety should prevail as first and foremost principle when looking after a patient with adrenal insufficiency who is exposed to major stress and we agree with Chee et al (1) that our pharmacokinetic data indicate that continuous hydrocortisone infusion is best suited to achieve prevention of adrenal crisis in this situation.

Chee et al. rightly enquired whether etomidate, an anesthetic agent that inhibits the crucial cortisol biosynthesis enzyme CYP11B1 (3), formed part of the anesthetic regimens that the patients with normal adrenal function received during elective surgery. We apologize that this information was hidden away in the supplementary information (Suppl. Table 1, (4)) and can confirm that, indeed, none of the patients received etomidate. Furthermore, acute or chronic intake of any drug known to impact on cortisol biosynthesis or metabolism during the last 6 months preceding the study procedures were exclusion criteria for study participation.

## Additional Information

***Disclosure Summary*:** All authors declare: no support from any organization for the submitted work other than that described above; no financial relationships with any organizations that might have an interest in the submitted work; no other relationships or activities that could appear to have influenced the submitted work.
